# Upper-Body Resistance Training and Self-Efficacy Enhancement in COPD

**DOI:** 10.4172/2161-105X.S9-001

**Published:** 2012-04-20

**Authors:** Margaret K. Covey, Edward McAuley, Mary C. Kapella, Eileen G. Collins, Charles G. Alex, Michael L. Berbaum, Janet L. Larson

**Affiliations:** 1 Department of Biobehavioral Health Science, College of Nursing, University of Illinois at Chicago, USA; 2 Department of Kinesiology and Community Health, College of Applied Health Sciences, University of Illinois at Urbana-Champaign, USA; 3 Department of Research and Development, Edward Hines Jr Veterans Administration Hospital, Chicago, USA; 4 Division of Pulmonary and Critical Care Medicine, Department of Medicine, Stritch School of Medicine, Loyola University, Chicago, USA; 5 Institute for Health Research and Policy, University of Illinois at Chicago, USA; 6 Acute, Critical and Long-Term Care Programs, School of Nursing, University of Michigan, USA

**Keywords:** Dyspnea, Exercise adherence, Muscle strength, Pulmonary rehabilitation

## Abstract

**Purpose:**

Loss of skeletal muscle strength is commonly seen with chronic obstructive pulmonary disease (COPD). The study aim was to determine the effects of comprehensive upper-body resistance training (8 different lifts) and a self-efficacy enhancing intervention in COPD with respect to muscle strength, symptoms, functional status and exercise adherence.

**Methods:**

This randomized trial had 3 groups: upper-body resistance training with an intervention to enhance self-efficacy (UBR + SE), upper-body resistance training and health education (UBR + HE), gentle chair exercises and health education (CE + HE). Subjects performed 16 weeks of supervised training, then 12 months of long-term maintenance at home. Outcomes were: muscle strength, dyspnea, functional status, self-efficacy, and adherence.

**Results:**

Sixty-four subjects completed 16 wks of training: age 71 ± 8 yr, fat-free mass index 19 ± 3 kg/m^2^, forced expiratory volume in one second 58 ± 18 percent predicted. The UBR + SE intervention produced a 46% increase in strength compared to a 36% increase in the UBR + HE group (*P* = 0.054). The combined UBR + SE and UBR + HE groups produced a 41% increase in strength compared to an 11% increase in the CE+HE (*P* < 0.001). The combined UBR groups also demonstrated increases in lean arm mass (*P* = 0.003) and a trend toward decreased dyspnea (*P* = 0.053). There were no group differences in attrition, attendance and training progression. Fifty subjects completed long-term maintenance and the UBR + SE and UBR + HE groups retained some gains in muscle strength, 24% and 21% respectively, and the CE + HE group lost 3% of muscle strength from baseline.

**Conclusion:**

The study provides strong evidence that comprehensive resistance training increased strength and lean arm mass and that strength can be partially maintained through a simple home program using hand weights. It provides limited evidence that upper-body resistance training improved dyspnea and that the exercise-specific self-efficacy enhancing intervention was beneficial.

## Introduction

Resistance training is employed in pulmonary rehabilitation to ameliorate the loss of skeletal muscle strength that is commonly seen in people with chronic obstructive pulmonary disease (COPD) [1,2]. Resistance training typically includes a combination of resistance exercises for upper-body, lower-body and trunk muscles with an emphasis on lower-body resistance training [1,2]. Little is known about the unique contribution of upper-body resistance training to outcomes such as symptoms and functional performance [3] and about long term maintenance after completion of a structured program of resistance training in people with COPD.

Differences in upper and lower extremity adaptations to COPD highlight the need to examine the separate effects of training each muscle group. There are differences in the morphological adaptations to COPD between the muscles of the upper body and lower body [3]. Greater fatigability has been observed for the upper extremity muscles. Additionally, the thoracoabdominal asynchrony that has been reported during unsupported arm activity [4] can contribute to intense dyspnea during activities involving the upper extremities. It was recently suggested that specific training protocols be developed for the different muscle groups [3].

Upper-body resistance training could potentially reduce dyspnea and improve capacity for arm activities by increasing strength of the arms, chest wall and respiratory muscles [5]. During upper-body resistance training, the muscles of the chest wall are used to stabilize the chest with each lift, potentially strengthening chest wall and respiratory muscles [6].

To optimize the effects of any training program it is important to address issues of exercise adherence [2,7]. Exercise adherence is a special problem for people with COPD because of daily fluctuation in symptoms. A variety of behavioral strategies have been used to promote exercise adherence in the general population and interventions to improve self-efficacy are among the most promising [8]. Self-efficacy, a belief in one’s ability to successfully carry out a course of action, has been consistently associated with adherence to exercise in the general population [9]. Those with a high sense of self-efficacy for exercise are more likely to engage in exercise, put forth more effort and persist over the long term despite setbacks such as daily fluctuation in symptoms. Self-efficacy is especially important during the initial adoption of exercise as individuals learn to overcome barriers to adherence [10], but it could also improve longer term adherence to exercise.

We examined short-term adoption and long-term maintenance effects of comprehensive upper-body resistance training and an exercise-specific self-efficacy intervention on upper-body strength, lean arm mass, dyspnea during physical activities, functional status, and on exercise-specific self-efficacy and exercise adherence in patients with COPD.

## Methods

### Subjects

Subjects with moderate to severe COPD [11] and no other major health problems were recruited. The inclusion criteria included: forced expiratory volume in one second (FEV_1_)/forced vital capacity < 70 and FEV_1_ < 80% predicted, age ≥ 45 years, currently in stable clinical condition (no exacerbations within two months of enrollment or recent change in medical therapy), and experienced dyspnea with upper-body activity. Screening procedures included: pulmonary function tests, arterial blood gases, physical examination, chest x-ray, resting electrocardiogram, Hospital Anxiety and Depression Scale [12], blood chemistries and hematology and urinalysis. Subjects who met eligibility criteria based on the above tests underwent a symptom-limited incremental cycle ergometer test. The research was approved by appropriate institutional review boards and all subjects gave written informed consent.

### Study design

This was a prospective randomized trial with one experimental and two control groups that performed 16 weeks of supervised training in the laboratory followed by 12 months of long-term maintenance, which consisted of unsupervised training at home. The experimental group received the combination of upper-body resistance training and an exercise-specific self-efficacy enhancing intervention (UBR+SE). One control group received resistance training with health education (UBR+HE), and the other control group received sham training (gentle chair exercise) with health education (CE+HE). Randomization to group was stratified by gender and disease severity (GOLD stages II, III, and IV) with a software program (biased coin algorithm to ensure equivalent groups) [13]. This was a concealed allocation process. Data collectors were blinded to the group assignment and subjects were not informed of the intent of the three group research design or the expected outcomes for each group. Measures of dependent variables were taken at baseline, after 4 months of supervised training, and at 6 and 12 months after the end of supervised training.

### Interventions

Subjects trained in small groups twice a week in the laboratory for 1.5 hours and once a week at home for 16 weeks. They were then instructed to train at home on their own for 12 months. During the long-term maintenance period subjects were seen for study visits at 3, 6, 9, and 12 months after the end of supervised training. All laboratory training was supervised by exercise specialists.

Resistance training in the laboratory was performed with a cable crossover system using 8 lifts: shoulder shrug, modified latissimus dorsi pulldown, overhead pulldown, front pulldown, front raise, upright row, biceps curl, and triceps extension. Training was initiated at 70% of the one repetition maximum (1RM), a measure of muscle strength, at a training volume of 2 sets of 8–10 repetitions. Training intensity was increased as tolerated to 80% of the 1RM over the first 4 weeks of training and adjusted to maintain an intensity of 80% of the current 1RM for the remaining weeks. Training volume was increased to 3 sets of 8–10 repetitions for weeks 5–16. For the home training subjects performed one set of 10–20 repetitions using dumbbells at a weight (range 2–15 lbs) to elicit a rating of perceived exertion equal to 12 (between light and somewhat hard) using the Borg Rate of Perceived Exertion scale (range 6–20) [14]. The lifts used for home sessions, engaged similar muscle groups compared to the supervised sessions. Subjects performed the home training once a week during the 16 weeks of supervised training and were instructed to perform home training three times a week on their own for the next 12 months.

Gentle chair exercises were a form of ‘sham’ training and incorporated stretching of all major joints and were based on the video “Armchair Fitness: Gentle Exercise” [15]. At home subjects performed similar chair exercises using a videotape.

The exercise-specific self-efficacy intervention was based on social cognitive theory [16] and closely modeled after self-efficacy interventions designed by Mc Auley and colleagues to promote adherence to an exercise program for middle-aged and older healthy adults [8,17]. During the 16 weeks of laboratory-based training, weekly interactive sessions (15 minutes) included overcoming barriers to exercise and maintaining exercise as a healthy life style. Subjects formed ‘buddy’ groups for support. They received structured feedback from staff and viewed videotapes of other people like themselves progressing through training. The self-efficacy program included guidance for returning to exercise after recovery from an exacerbation. During the 12 months of long-term maintenance subjects could remain in contact with their ‘buddy’ groups, but were not in contact with the staff in between scheduled study visits. Subjects attended a booster session at 3, 6 and 9 months after the end of supervised training. Each booster involved a visit to the laboratory that closely mimicked the earlier training sessions.

The health education program was modeled after a program used previously [18]. Sessions were conducted weekly during the 16 weeks of laboratory-based training and included respiratory physiology, medications and managing symptoms. During the long-term maintenance period sessions were conducted at 3, 6, and 9 months after the end of the supervised training on general health topics in place of the booster session subjects in the UBR+SE received in order to provide similar interaction with the research staff.

The fidelity of each intervention was established by periodic structured monitoring by objective observers. This was done to make sure that unique elements of each intervention were not inadvertently introduced into other groups.

### Outcome measures

Upper-body strength was assessed using the 1RM with the same 8 lifts used for supervised training. The 1RM represents the highest weight that can lifted one time over the full range of motion using proper lifting technique. Upper-body strength was reported as the sum of the 1RM for all 8 lifts. Inspiratory muscle strength was assessed with the maximal inspiratory pressure according to the technique of Black and Hyatt [19]. A practice session was conducted to familiarize subjects with the 1RM and the maximal inspiratory pressure. Lean arm mass was assessed with whole body dual-energy x-ray absorptiometry scans (Discovery Wi, Hologic Inc, Bedford, MA). Symptoms of dyspnea and fatigue were assessed with the Chronic Respiratory Disease Questionnaire (CRQ) [20]. The CRQ was administered during screening to familiarize subjects with the dyspnea scale prior to baseline measurements; at subsequent administrations subjects were not reminded of their previous responses.

Functional status was assessed with a performance-based test of upper-body function and the Functional Performance Inventory [21]. The Upper-Body Functional Performance Test was designed to simulate upper-body activities such as shelving groceries or dishes and involves arm work at various heights. Subjects moved four 0.9 kg bags filled with pellets through a series of five shelves with each lap starting at the bottom shelf, moving the bags (shelf-by-shelf) upward to the top shelf and then downward to the bottom shelf. Performance was reported as the number of completed laps within 5 minutes. The Functional Performance Inventory contains 67 items organized into eight subscales (body care, maintaining the household, physical exercise, recreation, spiritual activities, social interaction-family & friends, and work or school) which assess perceived difficulty in performing activities [21]. The instrument is scored as the mean of the items (range 0–3) for the total instrument.

Exercise-specific self-efficacy was assessed with self-report instruments: Self-Efficacy for Upper-Body Resistance Training Questionnaire (8 scales, one for each type of arm lift), Self-Efficacy for Upper-Body Physical Activity Questionnaire (4 scales, lifting objects, carrying objects, arm work such as mopping the floor or raking leaves, and arm work such as scrubbing or painting a wall), and the Barriers Efficacy Scale, COPD Version [22]. The Barriers Efficacy Scale measures people’s self-efficacy for overcoming barriers to exercise and three items were added to address issues specific to COPD (I felt short of breath when exercising, I felt tired, and I was sick). For the self-efficacy instruments, each item was rated on a scale of 0% (no confidence at all) to 100% (completely confident) and scores were calculated as the mean of the items. As self-efficacy is specific to the task, there were separate versions for the laboratory-based training period and the 12 months of long-term maintenance for both the Self-Efficacy for Upper-Body Resistance Training Questionnaire and the Barriers Efficacy Scale, COPD Version. Specifically the instructions were changed, directing subjects to indicate how self-confident they were that they could perform the exercises required during supervised training and long-term maintenance.

Three dimensions of adherence to the 16 weeks of laboratory-based training were examined: attrition (percent of subjects who withdrew from training for each group), attendance (percent of sessions attended by each subject) and the rate of progression for training intensity (change in the magnitude of weight lifted over the course of training for each subject). Attrition and attendance reflect the extent to which subjects showed up for participation in the training. Progression of training and strength gains reflect the extent to which subjects followed instructions and pushed themselves to perform during the training sessions. Adherence during the 12 months of long-term maintenance was assessed by retention of muscle strength; subjects who adhered to the exercise regimen would be more likely to retain muscle strength compared to those who did not.

### Lung Function

Comprehensive pulmonary function testing (lung volume measurements, spirometry, and diffusion capacity) was performed (VMAX Encore 22, Viasys Healthcare, Inc., Yorba Linda, CA) according to established standards [23].

### Data analysis

Intermittent missing data points were replaced using the last observation carried forward. Descriptive statistics are reported as mean + SD unless specified otherwise (SPSS version 14.0 for Windows, Chicago, IL). Baseline characteristics were examined with multivariate analysis of variance. Bivariate relationships were examined with Pearson correlations. Treatment outcomes were examined using multivariate analysis of variance for repeated measures with gender and COPD severity (FEV_1_ % predicted) as covariates. For the analysis of the supervised training phase of the study (*n* =64), 2 time points (baseline, immediately after 16 weeks supervised training) and all major outcome variables were included. For the analysis of the entire study period (*n* = 50), 4 time points (baseline, immediately after 16 weeks supervised training, 6 months after the end of supervised training, 12 months after the end of supervised training ) and fewer variables were included. The inclusion of fewer variables was necessary because dual energy x-ray absorptiometety was not performed at 6 months after supervised training and the versions of the self-efficacy instruments differed during the supervised and the unsupervised training phases of the study as described above. Statistical significance was set at *P* < 0.05. Where multivariate analysis of variance main effects were significant, ANOVA statistics for repeated measures were examined to determine differences between groups for individual variables (SPSS GLM LMatrix).

## Results

### Participants

**Figure 1:**
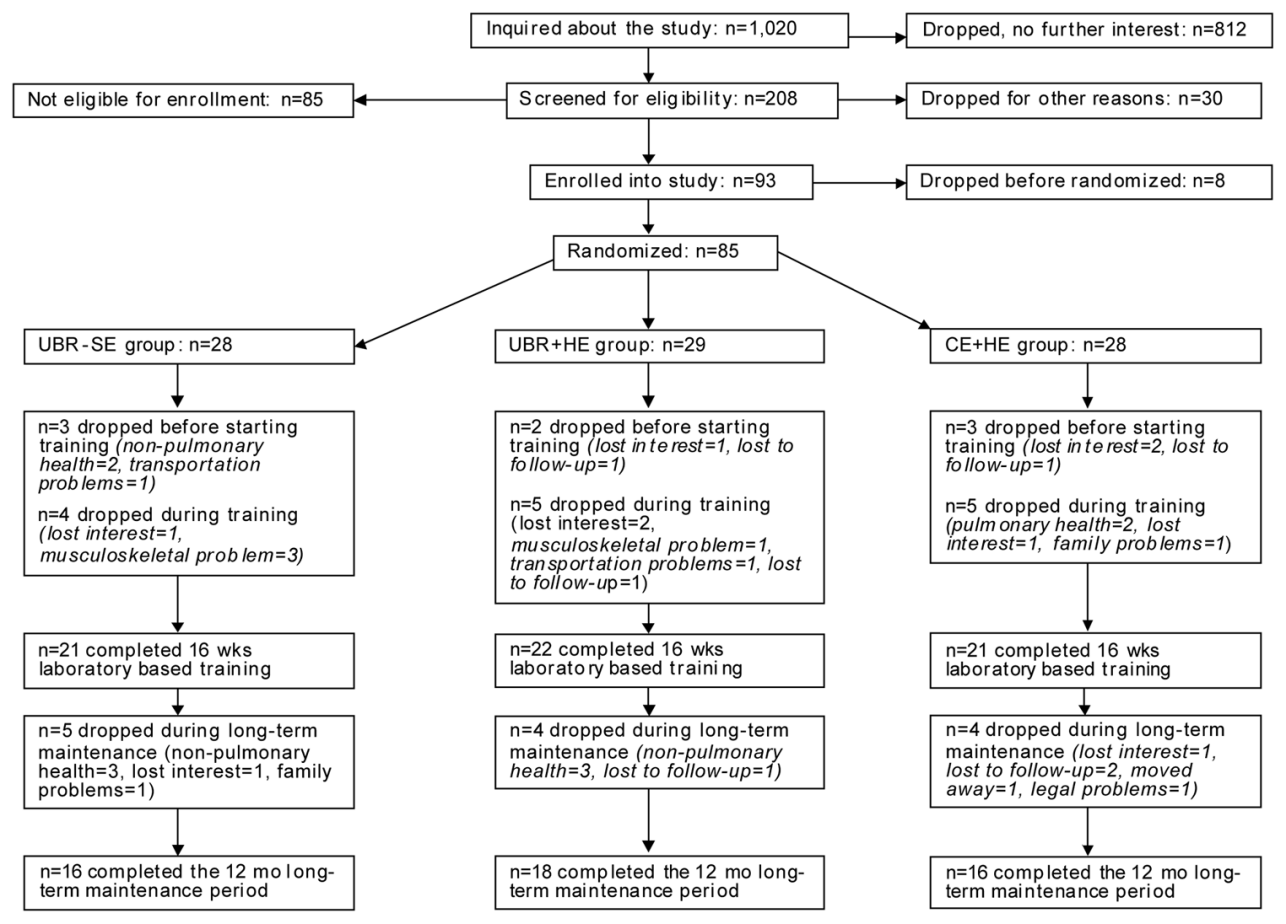
Flow of participants through the study.

Two hundred eight people were screened for eligibility, 93 enrolled, 77 initiated and 64 completed 16 weeks of laboratory-based training (*n* = 21 UBR + SE, n = 22 UBR + HE and n = 21 CE + HE); 50 went on to complete 12 months of long-term maintenance (n = 16 UBR + SE, n = 18 UBR + HE and *n* =16 CE + HE) (Figure 1). Attrition rates were similar between the 3 groups (*P* = 0.961); overall attrition was 17% during the 16 weeks of laboratory-based training, and an additional 22% withdrew during the 12 months of long-term maintenance. Those who dropped out during supervised training were not different from those who completed the supervised training with respect to age, body mass index, residual volume to total lung capacity ratio, but people who dropped out had greater airflow obstruction (FEV_1_ % pred: 39.8 + 16.9, *P* = 0.001) and lower diffusion capacity (single breath diffusing capacity % pred: 48.6 + 14.4, *P* = 0.019) (Table 1). The final sample included 50% GOLD II, 39% GOLD III, and 11% GOLD IV and fat-free mass was within normal for 84% of the sample [24].

### Sixteen weeks of supervised laboratory-based training

#### Training progression and attendance

**Figure 2:**
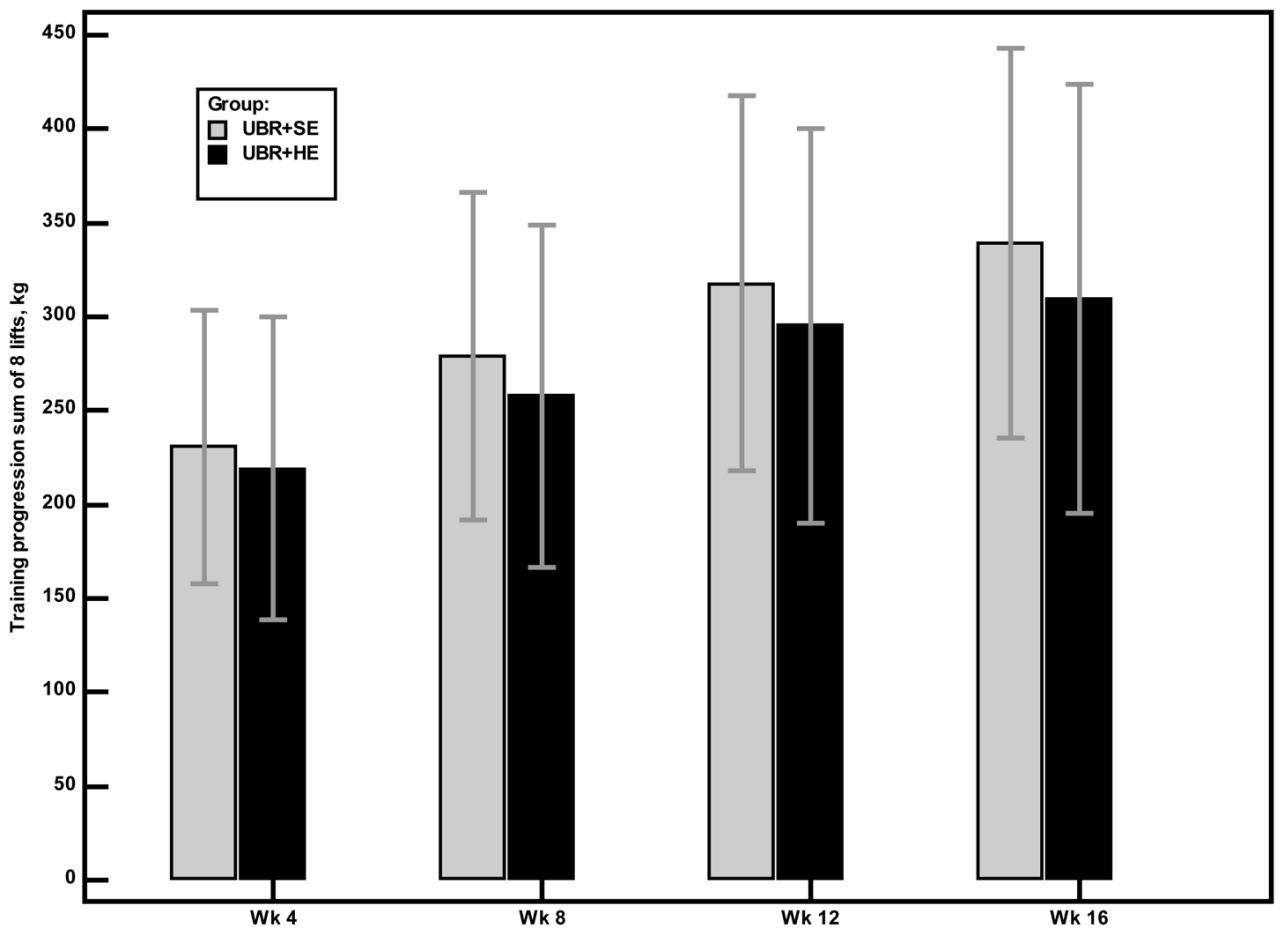
Training progression for latissimus dorsi pulldown lift for the UBR+SE and UBR+HE groups. Bars represent the mean values for the change in latissimus dorsi pulldown training loads.

Attendance rates were high in all 3 groups: 91% for UBR + SE, 89% for UBR + HE, and 91% for CE + HE groups (*P* = 0.722). Percentage of sessions attended was not related to baseline measures of age, disease severity, muscular strength, self-efficacy or lean arm mass. The most common reason for missing sessions was health reasons (30% of all absences) followed by vacation (20%), family responsibilities (12%), work (8%), musculoskeletal complaints (7%), and other miscellaneous reasons (23%). Training intensity increased over time (repeated measures effects, *P* < 0.001) in the two groups that performed resistance training and the rate of progression did not differ between groups (interaction effects (group × time), *P* = 0.101). Figure 2 describes the progression in training for the latissimus dorsi pulldown lift. Subjects tolerated the training well with no cardiovascular complications and few musculoskeletal injuries. Musculoskeletal events included muscle soreness (*n* =1), tendonitis in the elbow (*n* =1), exacerbation of an old shoulder injury (*n* =1); all but one completed the training.

#### Outcomes measures

There were no differences in training responses by gender or disease severity. The two resistance training groups demonstrated significant increases in upper-body strength, 46% and 36% increase from baseline for the UBR + SE and UBR + HE groups, respectively, compared to CE + HE (group × time, *P* < 0.001). There was a marginally greater improvement in upper-body strength for the UBR + SE group compared to the UBR + HE group, (interaction effect for the contrast, UBR + SE vs UBR+HE, *P* = 0.054). There were no other significant differences between the UBR + SE and UBR + HE groups, thus for subsequent analyses the two resistance training groups were pooled (UBR-Combined) to examine the effects of upper-body resistance training versus sham training (CE + HE) (Table 2).

Improvements in upper-body strength (UBR-Combined group 42 + 3 %; CE + HE group 12 + 17 %) and changes in lean arm mass (5 + 7%, and 0 + 5%, respectively) were significantly different with the UBR-Combined group demonstrating greater improvements than the CE+HE group (*P* < 0.001). Additionally, changes in strength were related to changes in lean arm mass (*r* = 0.50, *P* < 0.001). There were no changes in the Upper-Body Functional Performance test for either group.

The mean scores for CRQ Dyspnea improved by 12% in the UBR-Combined group with no change in the mean score for the CE + HE group (group × time interaction effect, *P* = 0.053). No changes were observed in CRQ Fatigue or the Functional Performance Inventory. Changes in CRQ Dyspnea were related to changes in CRQ Fatigue (*r* = 0.44, *P* < 0.001).

The UBR-Combined group demonstrated significant increases in self-efficacy for upper-body resistance training compared to the CE + HE group (group × time interaction, *P* < 0.001) and marginally significant increases in self-efficacy for performing upper-body physical activity compared to the CE + HE group (group × time interaction, *P* = 0.054). Changes in self-efficacy for upper-body resistance training were related to changes in 1RM (*r* = 0.44, *P* < 0.001), lean arm mass (*r* = 0.31, *P* = 0.012), and changes in self-efficacy for upper-body physical activities (*r* = 0.39, P=0.001). Changes in self-efficacy for performing upper-body physical activities were not significantly related to changes in strength (*r* = 0.23, *P* = 0.064). After 16 weeks of training there was no change in self-efficacy for overcoming barriers to exercise (Barriers Efficacy Scale, COPD Version) for either group.

### Long-term maintenance

**Figure 3:**
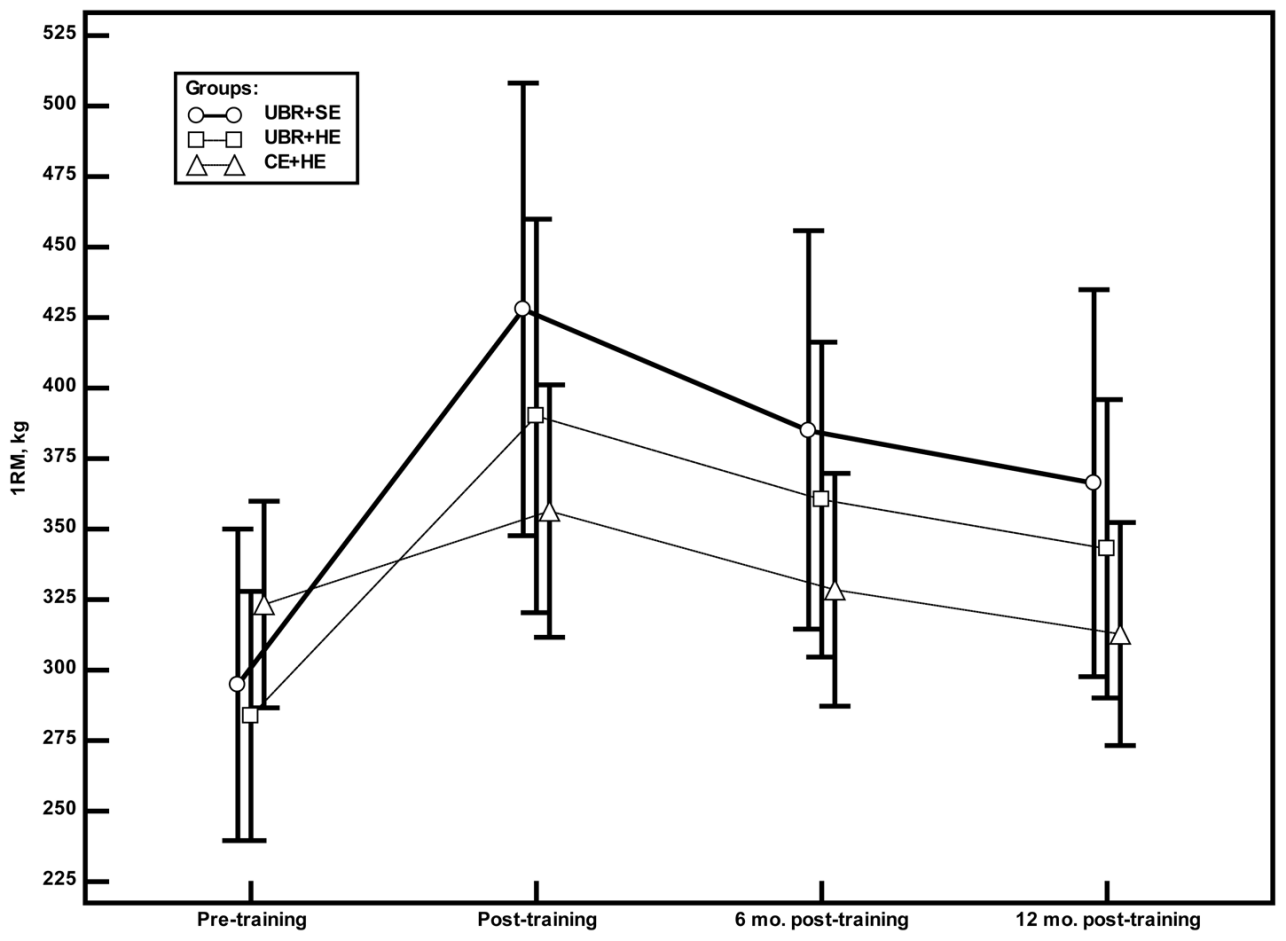
Changes in the 1RM by group. Symbols (○ =UBR+SE group, □ =UBR+HE group, △ =CE+HE group) represent the mean values for the 1RM.

There was a significant multivariate group × time interaction effect (multivariate analysis of variance, *P* = 0.045) for the three groups over the course of the study (baseline, 16 weeks of supervised training, 6 months after the end of supervised training, 12 months after the end of supervised training); however, only the univariate test of 1RM was significant (group × time, *P* = 0.001). Post hoc analysis comparing the baseline and 12 months post-supervised training time points revealed that the two resistance training groups maintained some of their gains in muscle strength (1RM was 24% and 21% above baseline value for the UBR + SE and UBR + HE groups respectively), whereas the CE + HE group lost 3% of their muscle strength compared to baseline (Figure 3). From the end of supervised training to 12 months after training, the 3 groups had similar changes in strength.

## Discussion

This is the first controlled trial of comprehensive upper-body resistance training in COPD that was not combined with pulmonary rehabilitation. Four months of supervised resistance training improved strength, lean arm mass and self-efficacy for upper-body resistance training with only marginally significant improvements in dyspnea during activities of daily life. Substantial strength gains were maintained for 12 months with an unsupervised home-based program. The exercise-specific self-efficacy enhancing intervention combined with resistance training provided only marginally significant improvements in acquisition of muscle strength compared to resistance training alone.

Observed improvements in muscle strength were similar in magnitude to healthy elderly subjects after upper-body resistance training [25] and to people with COPD who performed a combination of upper- and lower-body resistance training [1,26–29]. Theoretically, improvements in performance could result from real gains in strength or from improved muscle coordination. The observed increase in lean mass is consistent with real gains in strength and with previous studies of lower-body resistance training [30–32]. The strong relationship between the acquisition of muscle strength and lean mass further suggests these improvements were not merely the result of improved coordination. Note that subjects practiced the 1RM prior to baseline measurements to minimize the potential for learning effects. To our knowledge this is the first report of an increase in upper-body lean mass after resistance training in people with COPD.

The maintenance of substantial strength gains achieved during the supervised training suggests that adherence to 12 months of unsupervised home training was good. As the unsupervised home training was of lower intensity than the supervised training, we expected subjects would not completely retain all gains in strength achieved during the supervised portion of the program. Few studies have examined long term maintenance of resistance training in COPD [27,33,34]; of those only 2 followed upper-body strength after completion of the training program (12 weeks follow-up) and results were mixed, possibly owing to methodological differences [27,33]. Ortega et al. [27] found some decline in upper-body strength after 12 weeks (chest pull by 6%, butterfly by 18%, neck press by 10%), whereas O’Shea et al. [33] found that strength gains were not maintained 12 weeks after training. Both programs were of 12 weeks duration, but Ortega et al. [27] employed heavier resistance training (70–85% of the 1RM on a multigym) whereas O’Shea et al. [33] employed thera-bands. Data from the current study extends these findings by demonstrating that a simple unsupervised home training program can be maintained, sustaining considerable strength gains for up to 12 months after completion. By having subjects train at home once a week during the supervised portion of the program, subjects were confident and experienced with using the hand weights at home, which likely eased the transition from supervised to unsupervised training. This simple method could easily be implemented in an outpatient pulmonary rehabilitation setting.

Although behavioral interventions have been applied in other settings [17], there are no published data on the effects of a structured exercise-specific self-efficacy intervention in pulmonary rehabilitation. Our results are mixed. We observed a ceiling effect for attendance and attrition with very high attendance and low attrition for all groups, limiting the potential gains to be derived from the self-efficacy enhancing intervention. During the supervised training period, attendance rates compare favorably to data from other studies that examined the effects of resistance training in COPD [1,28–30,35]. In the current study, efficacy for overcoming barriers to exercise was high throughout the study and this could account for the high adherence during supervised training. Our results provide limited evidence that the self-efficacy enhancing intervention had a small effect on the acquisition of strength. Given the same rates of attendance this reflects adherence in terms of subjects’ willingness to exert themselves during training.

Unexpectedly, the gains in exercise-specific self-efficacy were similar for both resistance training groups. This cannot be explained by diffusion of the intervention between the two groups, because the integrity of the interventions was verified throughout the research to assure that the interventions were clearly different. This is likely explained by enactive mastery, the most powerful source of self-efficacy information [16]. Successes with training experienced by the UBR+HE group likely contributed to their improved confidence for performing the upper-body resistance training and upper-body activities in daily life. Having done it, they believed they could do it.

Gains in exercise-specific self-efficacy were modest. As suggested by McAuley et al. [36], it is conceivable that participants entering a randomized trial overestimate their capabilities because they have no real frame of reference upon which to formulate such judgements; this could dilute the measurable effects of the intervention. Our subjects were very sedentary with little prior exposure to resistance training.

Improvement in dyspnea during activities of daily life was marginally significant after resistance training. Three previous studies assessed the combined effects of upper- and lower-body resistance training alone (without pulmonary rehabilitation), and all found improvements in dyspnea after training [27–29]. However in a more recent study dyspnea did not improve [26]. Improvements in the CRQ Dyspnea scale were approximately 24% [27] and 36% [28]; whereas the improvement in dyspnea for the current study was approximately 12%. Subjects in the current study had less airflow obstruction; their maximal inspiratory pressure was relatively well preserved and it did not change with training, possibly explaining the smaller improvements in dyspnea. Additionally the use of a dyspnea scale that reflects general activities may have diluted the magnitude of effect for an intervention focused specifically on the upper-body. It is also important to note that on subsequent administrations of the CRQ we did not remind subjects of their previous answers as is commonly done with the CRQ dyspnea scale. This was to minimize the potential effect of subject bias toward improvement and it is a more rigorous test of effects on dyspnea when compared to studies that reminded subjects of their previous answers.

The lack of improvement in functional performance raises questions about the broader contribution of upper-body resistance training to functional outcomes. Previous research has been inconsistent with respect to functional outcomes [1]. We included subjects based on the presence of dyspnea during arm activities, not based on upper-extremity strength. Eighty four percent of the sample had a fat-free mass index within normal limits, which suggests these subjects had relatively preserved muscularity and strength which may have dampened the effects of the intervention on functional performance outcomes. Finally, the Functional Performance Inventory questionnaire targeted general activities, not just upper-body activities. We conclude that upper-body resistance training did not produce carryover effects to a broad array of daily activities.

Our findings and those of others suggest that resistance training is well tolerated by people with COPD [35]. The observed high adherence rates, low injury rates and strength gains support the feasibility of a comprehensive program for upper-body resistance training in COPD. It is important to give subjects active choices for exercise and comprehensive strength training could be included on the menu [37].

In summary, this study provides strong evidence that resistance training increased strength and lean arm mass and that strength can be partially maintained through a simple home program using hand weights. It provides limited evidence that resistance training improved dyspnea and that the exercise-specific self-efficacy enhancing intervention improved strength gains when combined with resistance training during supervised training. The self-efficacy intervention did not affect long-term maintenance.

## Figures and Tables

**Table 1:** Baseline Characteristics for Randomized Sample by Group.

	UBR+SE Group	UBR+HE Group	CE+HE Group
Gender ratio, male/female	17/4	17/5	18/3
Age, yrs	69.8 ± 9.0	71.0 ± 8.5	71.5 ± 7.5
BMI, kg/m2 *	30.1±7.4	26.2 ± 5.0	28.2 ± 6.4
FFMI, kg/m2	19.8 ± 3.5	18.1 ±2.9	19.1 ±2.3
FEV1, % predicted	59.4 ± 20.3	55.9±17.1	58.2 ± 16.0
RV/TLC, ratio	0.53±0.11	0.55±0.12	0.51 ± 0.08
DLco, % predicted	65.9 ± 21.4	64.7 ± 20.0	59.3 ± 22.2
HADS Depression Scale	4.3 ± 3.0	3.9 ± 3.0	3.8 ± 3.0

Data are presented as mean ± SD, with the exception of the gender ratio.

* Abbreviation: BMI: Body Mass Index; FFMI: Fat-free Mass Index; HADS: Hospital Anxiety and Depression Scale

MANOVA between-subjects effects: F (2,61) = 0.623, P = 0.072

* Using \/\/HO obesity guidelines [38] the sample was 1% underweight, 34% normal weight, 38% overweight, 11% grade 1 obesity, 11% grade 2 obesity, and 5% grade 3 obesity

**Table 2:** Outcome Measures Pre- and Post-Training Comparing UBR-Combined and CE+HE Groups.

	Group	Baseline	Week 16	Group X time effects*
1RM-sum of 8 lifts, kg	UBR-Combined CE+HE	290±98313±78	409 ± 149350 ± 90	P < 0.001
Whole body fat, %	UBR-Combined CE+HE	30.3 ± 7.129.3 ± 7.4	29.6 ± 7.329.5 ± 7.4	P = 0.024
Lean arm mass, kg	UBR-Combined CE+HE	6.1 ± 1.86.1 ± 1.1	6.4 ± 1.9 6.1 ± 1.2	P = 0.003
Plmax, cm H2O	UBR-Combined CE+HE	76.6 ± 26.2 75.6 ± 23.6	75.7 ± 25.8 71.5 ± 24.8	P=0.166
CRQ Dyspnea, scale score	UBR-Combined CE+HE	4.1 ± 1.04.2 ± 1.1	4.6 ±1.2 4.2 ± 1.1	P = 0.053
CRQ Fatigue, scale score	UBR-Combined CE+HE	4.2 ± 1.14.4 ± 1.0	4.8 ± 1.2 4.9 ± 1.0	P=0.145
UB-FPT, laps	UBR-Combined CE+HE	7.1 ± 1.66.8 ± 2.1	7.2 ±1.7 7.2 ± 2.0	P=0.194
FPI, total score	UBR-Combined CE+HE	2.1 ±0.42.1 ±0.5	2.2 ± 0.5 2.2 ± 0.5	P = 0.316
SE-Upper Body Resistance Training, total score	UBR-Combined CE+HE	49±2149±22	75±1853±23	P < 0.001
SE-Upper Body Physical Activity, total score	UBR-Combined CE+HE	58 ± 1858 ± 25	66 ± 20 60 ± 23	P = 0.054
BE-COPD, total score	UBR-Combined CE+HE	75±1877± 18	75 ± 2270 ± 26	P=0.137

Data are mean ± SD.

* Abbreviations: BE-COPD: Barriers Efficacy COPD Version; FPI: Functional Performance Inventory; PIMAX: Maximal Inspiratory Pressure; SE-Upper-Body Exercise: Self-Efficacy for Upper-Body Exercise; SE-Upper Body Physical Activity: Self-Efficacy for Upper-Body Physical Activity; UB-FPT: Upper-Body Functional Performance Test MANOVA between-subjects: Group F(1,62) = 0.739, P = 0.697; gender F(1,62) = 8.707, P < 0.001; FEV1 % predicted F(1,62) = 3.664, P = 0.001.

MANOVA within-subjects effects: time F(1,62) = 0.709, P = 0.724; interaction of time by group assignment F(1,62) = 3.720, P = 0.001; interaction of time by gender F(1,62)=1.165, P = 0.335; interaction of time by disease severity (FEV1 % predicted) F(1,62) = 0.714, P = 0.719 (MANOVA P value is 2-tailed).

* Individual ANOVA P value is one-tailed.

## Abbreviations

1RM: One Repetition Maximum
CE: Chair Exercise
COPD: Chronic Obstructive Pulmonary Disease
CRQ: Chronic Respiratory Disease Questionnaire
FEV_1_: Forced Expiratory Volume in One Second
HE: Health Education
SE: Self-Efficacy
UB: Upper-Body
UBR: Upper-Body Resistance Training
